# Combined chlorogenic acid and baicalin supplementation alleviates PRRSV-induced disease by modulating immune responses and gut microbiota in weaned piglets

**DOI:** 10.1128/spectrum.01602-26

**Published:** 2026-09-28

**Authors:** Li He, Shao-shi Ji, Ying Guan, Bao-Ling Liu, Bing-Bing Huang, Tao Liu, Zhi-wen Xu, Ling Zhu

**Affiliations:** 1 College of Veterinary Medicine, Sichuan Agricultural University 506176, Chengdu, China; 2 Jiangxi Academy of Sciences 230144, Nanchang, China; 3 Haotian Pharmaceutical Co., Ltd., Zhucheng City, China; 4 College of Veterinary Medicine Sichuan Key Laboratory of Animal Epidemic Disease and Human Health, Sichuan Agricultural University 506176, Chengdu, China; Shandong First Medical University, Jinan, Shandong, China

**Keywords:** porcine reproductive and respiratory syndrome virus, chlorogenic acid, baicalin, immune response, gut microbiota, weaned piglets

## Abstract

Porcine reproductive and respiratory syndrome continues to pose a major challenge to the global swine industry, leading to substantial economic losses, and complicating disease management. This study evaluated whether dietary supplementation with chlorogenic acid and baicalin could alleviate clinical disease, modulate inflammatory and antiviral immune responses, and reshape the gut microbiota in porcine reproductive and respiratory syndrome virus (PRRSV)-infected weaned piglets. Forty-eight weaned male piglets were randomly assigned to four groups: negative control, PRRSV-infected positive control, low-dose treatment, and high-dose treatment. Piglets in the PC, LG, and HG groups were challenged with PRRSV, whereas the NC group received sterile PBS. During the experiment, the clinical manifestations of the piglets were recorded in detail. At 21 dpi, samples were collected to evaluate immune responses and gut microbiota composition. Compared with the PC group, the HG group showed only a mild and transient increase in rectal temperature, peaking at 3 dpi and returning to the normal range by 6 dpi, increased body weight by 21.0% at 21 dpi, reduced serum PRRSV RNA by 1.18 log_10_ copies/mL at 7 dpi, and decreased serum viral RNA to below the qPCR detection limit by 21 dpi. HG treatment also decreased TNF-α, IL-1β, and IL-6 by 26.1–40.9% and increased IFN-γ and IL-2 by 17.8–151.1% at day 21 dpi with PC. Microbiota analysis showed increased Lactobacillaceae abundance and reduced opportunistic taxa, including Clostridiaceae, Peptostreptococcaceae, and Staphylococcaceae. These findings suggest that combined chlorogenic acid and baicalin supplementation may serve as a promising adjunctive nutritional strategy for mitigating PRRSV-associated disease in weaned piglets. Further studies using contemporary field strains and long-term production trials are needed to validate its practical efficacy and safety.

**IMPORTANCE:**

Porcine reproductive and respiratory syndrome virus (PRRSV) causes severe economic losses in pig production and remains difficult to control because of viral variability and incomplete vaccine protection. This study shows that a chlorogenic acid-baicalin combination can reduce PRRSV-induced clinical disease, promote viral clearance, regulate inflammatory and antiviral immune responses, protect organs, and restore gut microbiota in infected piglets. These findings highlight the potential of plant-derived bioactive compounds as sustainable, antibiotic-free interventions for PRRSV control. The work provides experimental evidence supporting the development of natural functional feed additives to improve animal health and reduce reliance on conventional antimicrobial strategies in modern swine production.

## INTRODUCTION

Porcine reproductive and respiratory syndrome virus (PRRSV) is a single-stranded RNA virus that primarily infects the respiratory and reproductive systems of pigs. Since its first discovery in the 1980s, PRRSV has spread globally, becoming one of the most devastating pathogens in the swine industry (1, 2). The virus can be transmitted via airborne, direct contact, and vertical (mother-to-offspring) routes. Infection typically leads to clinical signs such as high fever, labored breathing, reduced appetite, and diarrhea, and can even result in death in severe cases (1, 3). PRRSV also induces reproductive disorders including miscarriages and stillbirths, significantly impairing sow reproductive performance. Because of its diverse transmission routes and high virulence, controlling PRRSV remains a major challenge for the global pig industry and has caused enormous economic losses (4).

The intestinal microbiota plays a pivotal role in maintaining pig health and regulating immunity, especially during PRRSV infection, as its balance directly affects the host’s immune response and disease resistance (5). A healthy microbiota interacts with immune cells to maintain immune homeostasis and induces the secretion of anti-inflammatory cytokines (e.g., IL-10), thereby preventing excessive inflammation (6). An intact gut barrier and mucosal immunity can reduce viral invasion. However, PRRSV often causes microbiota dysbiosis: beneficial bacteria (such as Lactobacillus and Bifidobacterium) decrease, while pathogenic bacteria (like Klebsiella and Proteus) increase, exacerbating immunosuppression and inflammatory responses (7, 8). Therefore, maintaining gut microbial diversity not only helps alleviate clinical symptoms but also accelerates viral clearance by activating immune cells and strengthening the mucosal barrier (5, 7, 8).

Chlorogenic acid (CGA) is a natural phenolic acid widely found in plants such as coffee, goji berries, and apples, and it exhibits multiple bioactivities including antiviral, anti-inflammatory, antioxidant, and immunomodulatory effects. Previous studies have reported that CGA may exert antiviral effects by interfering with viral attachment, entry, and replication. It has also been associated with reduced production of pro-inflammatory cytokines, such as TNF-α and IL-6, and modulation of inflammation-related pathways, including NF-κB and MAPK (9, 10).

Baicalin (BAI), a flavonoid compound extracted from the traditional Chinese herb Scutellaria baicalensis (Huangqin), likewise demonstrates notable antiviral, anti-inflammatory, antioxidant, and immunomodulatory capacities (11–13). Previous studies suggest that these effects may be related to modulation of viral entry, replication, and inflammatory mediator production.

According to traditional Chinese medical literature, many herbal formulas containing CGA and BAI are renowned in clinical practice for their heat-clearing, detoxifying, anti-inflammatory, and antiviral properties. Traditional Chinese medicine formulations containing CGA- or BAI-related herbal components, such as Yin-Qiao San and Huang-Lian Jie-Du Tang, have long been used for heat-clearing and detoxifying purposes (14, 15). This empirical evidence provides a historical basis for the combined use of these two components.

Previous studies have independently reported antiviral, anti-inflammatory, antioxidant, and immunomodulatory activities of CGA and BAI (12, 16). However, the effects of their combined administration in PRRSV-infected piglets remain insufficiently characterized. Therefore, the present study evaluated whether combined dietary supplementation with CGA and BAI could alleviate clinical disease, modulate inflammatory and antiviral immune responses, reduce serum PRRSV RNA load, and reshape the gut microbiota in PRRSV-infected weaned piglets.

## MATERIALS AND METHODS

### Animals and virus

Forty-eight 21-day-old weaned male piglets (Landrace × Large White, body weight 5.0–7.0 kg) were selected, all from the same pig farm. The experimental piglets were strictly grouped and isolated to ensure physical separation between groups, with dedicated housing rooms and areas. Environmental conditions, including temperature, humidity, ventilation, and lighting, were carefully controlled. Staff wore protective clothing, footwear, and gloves, and adhered to biosafety protocols. Daily cleaning and disinfection were conducted, and separate water and feed were provided for each group. Regular health monitoring was performed to prevent cross-contamination. Physical and molecular examinations confirmed that all piglets were free of parasitic, respiratory, or gastrointestinal viral diseases. At 28 days of age, the piglets had an immature immune system, making them highly susceptible to PRRSV and thus suitable as infection models. The PRRSV strain used in this study was CH/SCGY-2/2020, a China-origin PRRSV-2 strain with GenBank accession number MZ747438.1. This strain was selected because it represents a locally relevant PRRSV strain maintained in our laboratory and had been confirmed in preliminary observations to induce typical PRRSV-associated clinical signs and pathological lesions in weaned piglets, including fever, respiratory distress, reduced appetite, and lung injury. Therefore, it was considered suitable for establishing a reproducible PRRSV infection model in weaned piglets. The virus was propagated in MARC-145 cells and titrated as 50% tissue culture infectious doses (TCID_50_) using the Reed–Muench method.

### Main reagents and consumables

All experiments employed analytical-grade reagents and certified consumables: CGA (≥98% purity; Chengdu Must Bio-Technology Co., Ltd., Chengdu, China) and BAI (≥ 98% purity; ChemFaces Biochemical Co., Ltd., Wuhan, China) were used without further purification; PRRSV RNA quantification was carried out using the RealPCR PRRS Types 1-2 Multiplex RNA Mix real-time RT-PCR kit (IDEXX Laboratories, Westbrook, ME, USA) according to the manufacturer’s protocol; serum levels of TNF-α, IL-1β, IL-6, IFN-γ, IL-2, and IL-10 were determined using porcine ELISA kits (R&D Systems, Minneapolis, MN, USA) according to the manufacturer’s instructions; microbial DNA was extracted from colonic contents using the FastPure Stool DNA Isolation Kit (MiaoJinYuan, Shanghai, China) and amplified with FastPfu Polymerase (TransGen Biotech, Beijing, China) on a T100 PCR thermocycler (Bio-Rad, Hercules, CA, USA); electrophoretic analysis was performed on a JY600C system (Junyi Dongfang, Beijing, China); and high-throughput sequencing was conducted by Shanghai Majorbio Bio-Pharm Technology Co., Ltd. All pipette tips, centrifuge tubes, PCR tubes, and related consumables were sourced from manufacturers adhering to stringent laboratory quality standards.

### Infection model establishment

After a 1-week acclimation period, piglets in the PC, LG, and HG groups were intranasally inoculated at 0 dpi with 1 mL of PRRSV suspension containing 1 × 10^6^ TCID_50_, whereas piglets in the negative control (NC) group received 1 mL of sterile PBS by the same route. The day of PRRSV challenge was designated as 0 dpi. Blood samples were collected at −7, 0, 7, 14, and 21 dpi. Rectal temperature was recorded daily from −7 to 21 dpi.

### Experimental design

The piglets were randomly assigned to four experimental groups (*n*  =  12 per group), including a negative control (NC) group, a positive control (PC) group (PRRSV), a low-dose treatment group (LG, PRRSV + the low dose of CGA and BAI combination), and a high-dose treatment group (HG, PRRSV + the high dose of CGA and BAI combination). From 0 through 21 dpi, CGA and BAI were mixed into the feed once daily at the designated doses. The dosage was adjusted every 7 days based on body weight (measured weekly) to ensure accurate administration. The specific dosing regimens for each group are detailed in Table 1. The doses of CGA and BAI were selected based on previous studies in weaned piglets and preliminary tolerance observations, in which supplementation within this range showed intestinal protective and immunomodulatory effects without apparent adverse reactions. The low dose was used to assess basal efficacy, whereas the high dose was designed to determine whether increased exposure enhanced antiviral and anti-inflammatory responses.

**TABLE 1:** Dosage regimen for CGA and BAI in each group

	Negative control (NC)	Positive control (PC)	Low-dose group (LG)	High-dose group (HG)
CGA	0	0	50 mg/kg BW/day 0–21 dpi	100 mg/kg BW/day 0–21 dpi
BAI	0	0	50 mg/kg BW/day 0–21 dpi	100 mg/kg BW/day 0–21 dpi
Challenge at 0 dpi	1 mL sterile PBS	1 mL 1 × 10^6^ TCID_50_	1 mL 1 × 10^6^ TCID_50_	1 mL 1 × 10^6^ TCID_50_

### Clinical monitoring and sampling

Clinical signs were recorded daily as described in “Experimental design” section. Blood samples were collected at −7, 0, 7, 14, and 21 dpi. Serum PRRSV RNA was quantified at 7, 14, and 21 dpi. Serum cytokines were measured at 0, 7, 14, and 21 dpi. At 21 dpi, piglets were euthanized, and colonic contents were collected. DNA was extracted using the FastPure Stool DNA Isolation Kit according to the manufacturer’s instructions. DNA integrity was verified by 1% agarose gel electrophoresis, and concentration and purity were measured with a NanoDrop 2000 spectrophotometer.

### Histopathological scoring

Histopathological lesions were evaluated using a semi-quantitative 0–4 ordinal scale based on the severity and extent of tissue-specific lesions in H&E-stained sections. Scores were defined as follows: 0, no obvious lesion; 1, minimal lesion; 2, mild lesion; 3, moderate lesion; and 4, severe lesion. For each piglet, three randomly selected non-overlapping microscopic fields from each organ were evaluated. The mean of the three field scores was used as the final lesion score for that organ. All sections were evaluated by a veterinary pathologist who was blinded to the treatment groups.

### PCR amplification and library construction

The V3–V4 region of the 16S rRNA gene was amplified using 338F/806R primers with barcodes (17) under the following conditions: 95°C for 3 min; 27 cycles of 95°C for 30 s, 55°C for 30 s, 72°C for 30 s; and a final extension at 72°C for 10 min. PCR products were purified from a 2% agarose gel and then used to construct libraries with the NEXTFLEX Rapid DNA-Seq Kit, followed by sequencing on an Illumina NextSeq 2000 platform.

### High-throughput sequencing analysis

Quality control was performed using fastp (18), and paired-end sequences were merged with FLASH (19). Denoising and removal of chloroplast/mitochondrial sequences were done with QIIME2-DADA2 (20). After rarefying to 20,000 reads per sample, taxonomic assignment was carried out against the SILVA database (21).

### Statistical analysis

Longitudinal data, including rectal temperature, body weight, serum PRRSV RNA load, and cytokine concentrations, were analyzed using two-way repeated-measures ANOVA, with treatment group as the between-subject factor, time as the within-subject factor, and group × time interaction included in the model. When significant main effects or interactions were detected, Tukey’s multiple-comparison test was used for post hoc comparisons. Data were assessed for normality and sphericity; when the sphericity assumption was violated, the Greenhouse–Geisser correction was applied. If missing values were present or repeated-measures assumptions were not satisfied, a mixed-effects model was used. Data are presented as mean ± SD, and *P* < 0.05 was considered statistically significant.

Microbiota analyses were performed on the Majorbio Cloud Platform. Alpha diversity indices, including Chao1 and Shannon indices, were calculated using mothur (22). Differences in alpha diversity among groups were assessed using the Kruskal–Wallis test, followed by pairwise Wilcoxon rank-sum tests where appropriate. Community structure was visualized by principal coordinate analysis (PCoA). Histopathological lesion scores were treated as ordinal data and analyzed using the Kruskal–Wallis test, followed by Dunn’s multiple-comparison test.

## RESULTS

### Clinical presentation of piglets

**Fig 1:**
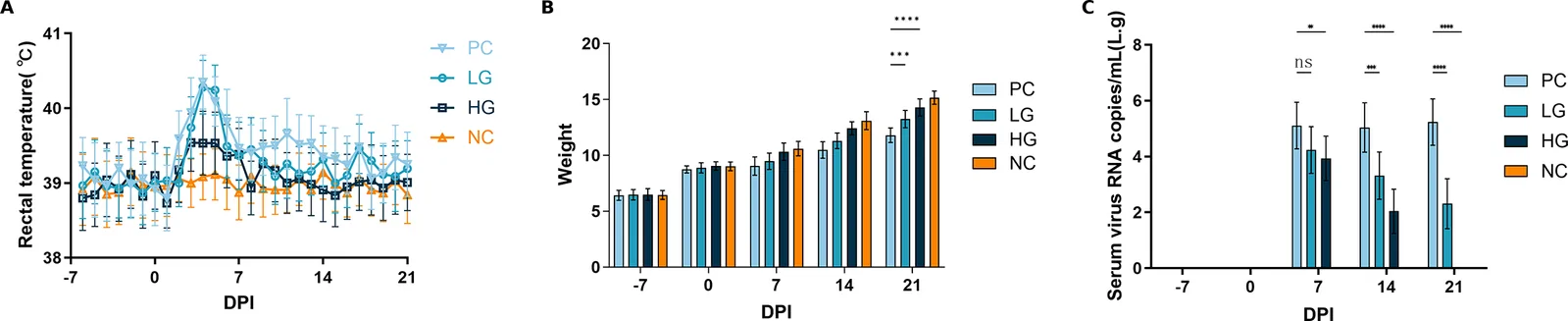
Effects of CGA plus BAI on rectal temperature, body weight, and serum. PRRSV challenge was performed at 0 dpi; −7 to −1 dpi represented the baseline period. (**A**) Time course of rectal temperature from −7 to 21 dpi. (**B**) Body weight recorded at −7, 0, 7, 14, and 21 dpi. (**C**) Serum PRRSV RNA load measured at 0, 7, 14, and 21 dpi. Groups: NC, negative control; PC, positive control; LG, low-dose group; HG, high-dose group. Longitudinal data were analyzed using two-way repeated-measures ANOVA with treatment group, time, and group × time interaction as factors, followed by Tukey’s multiple-comparison test for post hoc comparisons. Statistical annotations indicate pairwise comparisons between groups at the indicated time points: ns, not significant; *P < 0.05; **P < 0.01; ***P < 0.001; and ****P < 0.0001.

Rectal temperature changes (Fig. 1A): after PRRSV challenge, the PC group developed a sustained febrile response, with mean rectal temperature exceeding 39.5°C from 2 to 5 dpi post-infection and reaching a peak of 40.35 ± 0.36°C at 4 dpi. In contrast, the LG group showed a less severe fever response, whereas the HG group exhibited only a mild and transient increase, peaking at 39.54 ± 0.38°C at 3 dpi and returning to the normal range by 6 dpi. The NC group maintained rectal temperatures within the normal range throughout the experiment. Two-way repeated-measures ANOVA showed significant effects of treatment group, time, and group × time interaction (all *P* < 0.0001), indicating that high-dose CGA–BAI treatment significantly attenuated PRRSV-induced fever and accelerated recovery toward normothermia.

Body weight and clinical signs (Fig. 1B): PRRSV-infected piglets in the PC group showed reduced appetite, depression, respiratory signs, and impaired weight gain. These clinical abnormalities were milder in the LG group and were markedly attenuated in the HG group. At 21 dpi, body weight was 11.80 ± 0.66 kg in the PC group, 13.24 ± 0.78 kg in the LG group, and 14.28 ± 0.77 kg in the HG group. Compared with the PC group, HG treatment increased final body weight by 21.0%, suggesting that high-dose CGA–BAI supplementation alleviated clinical disease and improved growth performance after PRRSV infection.

### Serum PRRSV RNA load

At 7 dpi, serum PRRSV RNA levels in the HG group were significantly lower than those in the PC group, decreasing from 5.11 ± 0.83 to 3.93 ± 0.80 log_10_ copies/mL, corresponding to a reduction of 1.18 log_10_ copies/mL (*P* < 0.01). By 21 dpi, serum PRRSV RNA in the HG group had decreased to below the qPCR detection limit, whereas the PC group remained viremic at 5.23 ± 0.83 log_10_ copies/mL (Fig. 1C).

### Serum cytokines

**Fig 2:**
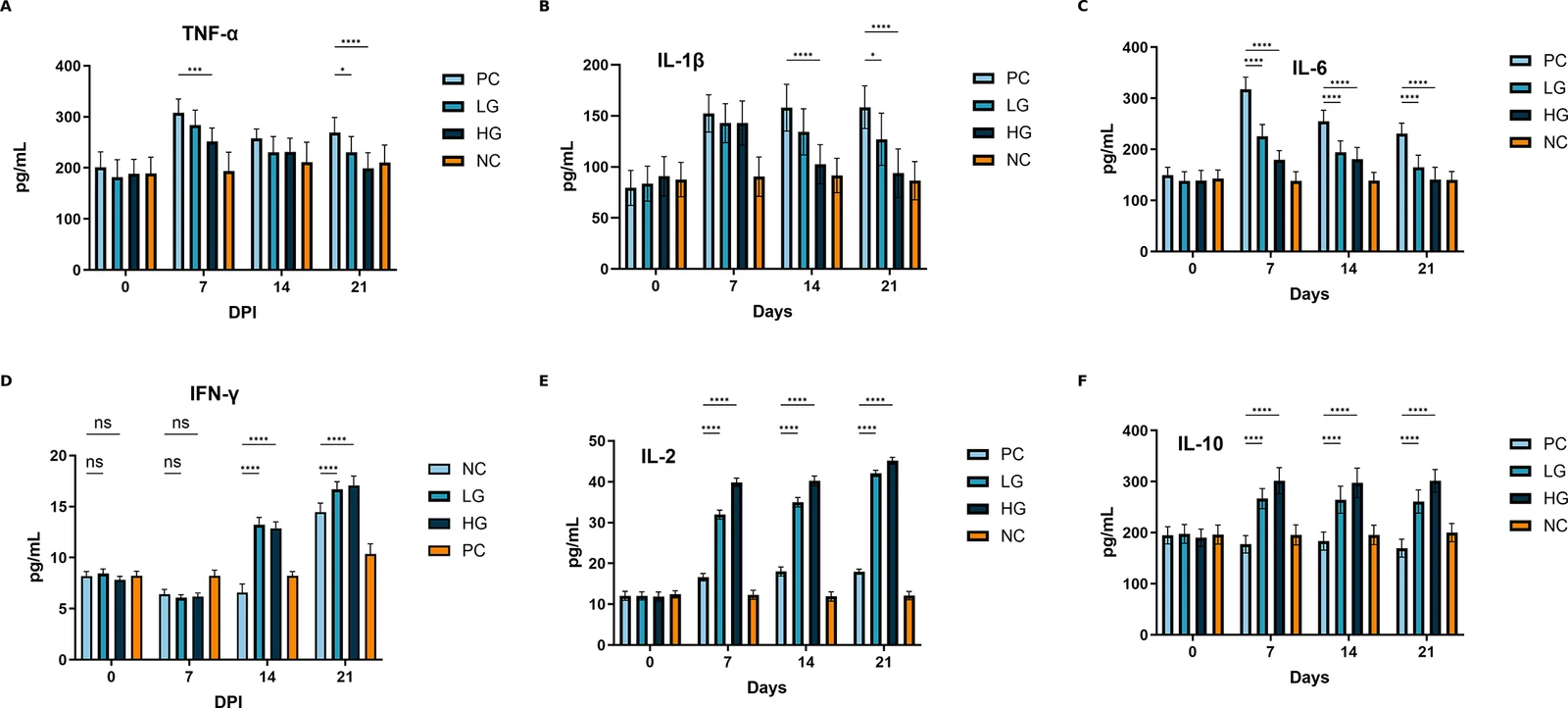
Dynamic profiles of serum cytokines. Serum cytokine concentrations were measured at 0 dpi immediately before PRRSV challenge and at 7, 14, and 21 dpi. (**A**) TNF-α, (**B**) IL-1β, (**C**) IL-6, (**D**) IFN-γ, (**E**) IL-2, and (**F**) IL-10. Group designations: NC, negative control; PC, positive control; LG, low-dose group; HG, high-dose group. Data were analyzed using two-way repeated-measures ANOVA or mixed-effects models, followed by Tukey’s multiple-comparison test: ns, not significant; *P < 0.05; ***P < 0.001; and ****P < 0.0001.

Compared to the NC group, the PC group exhibited persistently elevated levels of TNF-α (Fig. 2A), IL-1β, and IL-6 throughout the experiment, along with relatively lower levels of IFN-γ and IL-2. Starting at 7 dpi, serum TNF-α, IL-1β, and IL-6 concentrations in the LG and HG groups were significantly lower than those in the PC group (*P*  <  0.05). IL-2 concentrations increased significantly from 7 dpi, whereas a significant increase in IFN-γ was observed at 14 dpi and became more pronounced at 21 dpi. IL-10 concentrations became significantly higher in the LG and HG groups than in the PC group beginning at 7 dpi. Overall, the HG group showed a greater magnitude of reduction in pro-inflammatory cytokines and increase in antiviral cytokines than the LG group, suggesting that the high-dose combination effectively suppressed inflammation while more robustly activating the antiviral immune response (Fig. 2).

###  Histopathology

**Fig 3:**
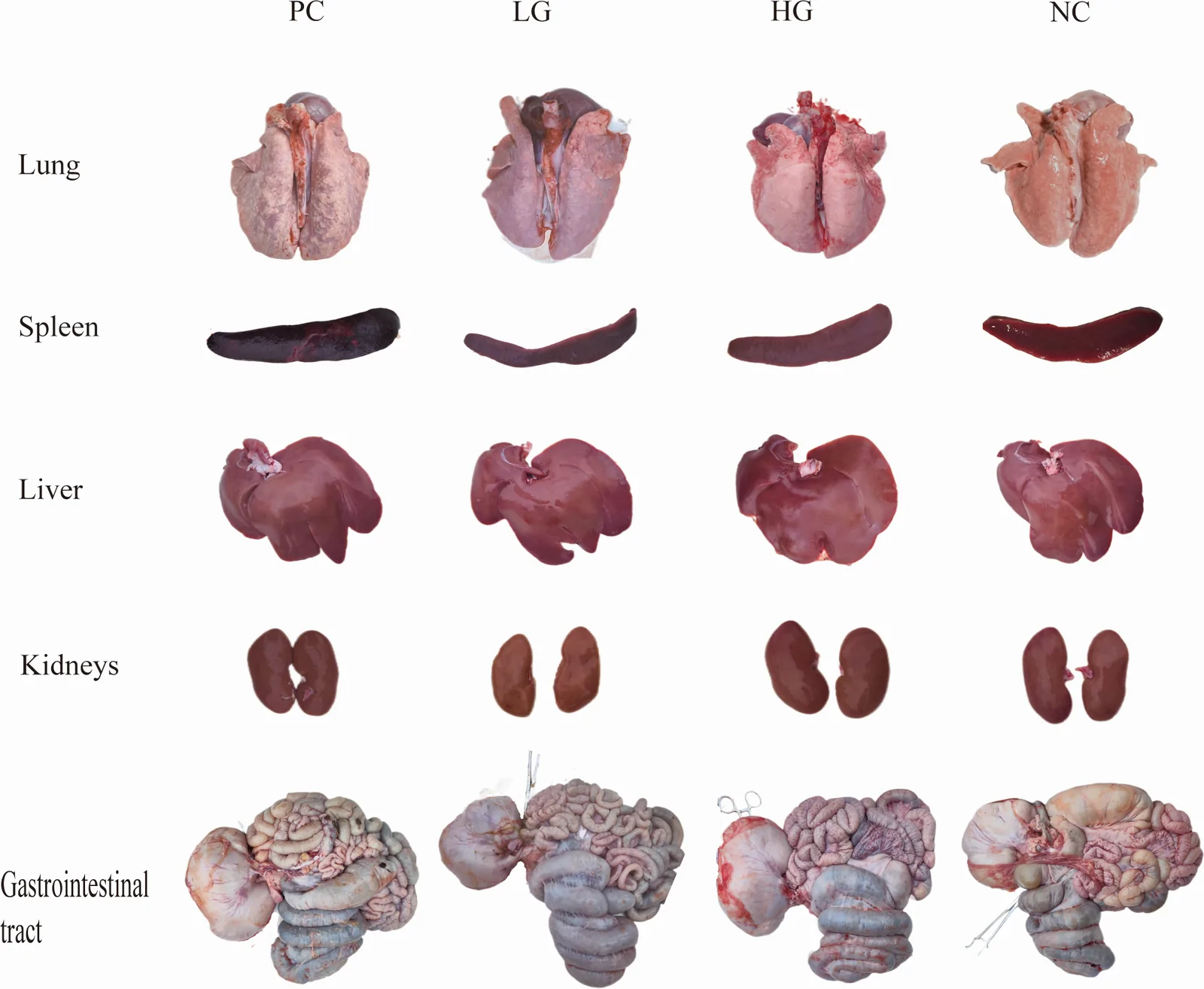
Macroscopic lesions of major organs. Representative macroscopic photographs of lung, spleen, liver, kidneys, and gastrointestinal tract collected at 21 dpi. Organs are shown from left to right for the positive control (PC), low-dose (LG), high-dose (HG), and negative control (NC) groups. Severe congestion, consolidation, and enlargement are evident in PC, partially alleviated in LG, markedly reduced in HG, and absent in NC.

Gross pathological examination at 21 dpi revealed marked PRRSV-associated lesions in the PC group, including pulmonary consolidation or “hepatization,” congestion, hemorrhage, edema, and enlargement of multiple organs. These gross lesions were partially alleviated in the LG group and were markedly reduced in the HG group, in which only mild pulmonary congestion was observed. No obvious gross abnormalities were detected in the NC group (Fig. 3).

**Fig 4:**
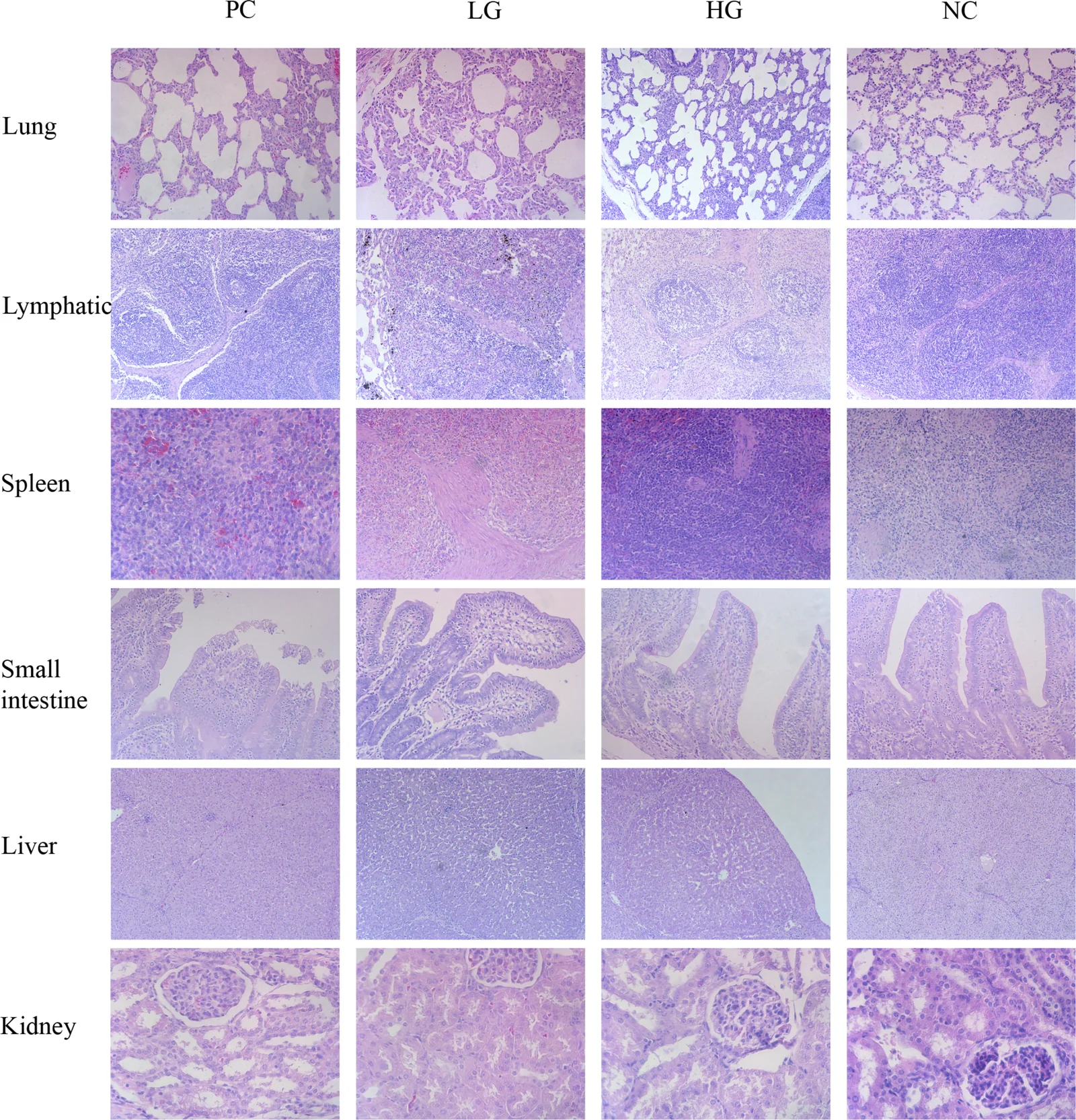
Histopathological changes, hematoxylin and eosin (H&E) stain. Hematoxylin and eosin-stained sections of lung, lymph node, spleen, small intestine, liver, and kidney collected at 21 dpi (original magnification ×100). Columns represent PC, LG, HG, and NC groups; rows correspond to the indicated organs. Extensive alveolar damage, lymphoid depletion, splenic necrosis, villus atrophy, hepatocellular necrosis, and renal tubular lesions were observed in the PC group, attenuated in the LG and HG groups, and absent or minimal in the NC group.

Histopathological examination further confirmed PRRSV-induced multi-organ injury. The PC group showed severe pathological changes, including destruction of alveolar architecture, lymphoid depletion, splenic lesions, villus atrophy or intestinal mucosal shedding, hepatocellular degeneration or necrosis, and renal tubular injury. Tissue damage was moderately reduced in the LG group and was further attenuated in the HG group, whereas no obvious histopathological abnormalities were observed in the NC group (Fig. 4).

**Fig 5:**
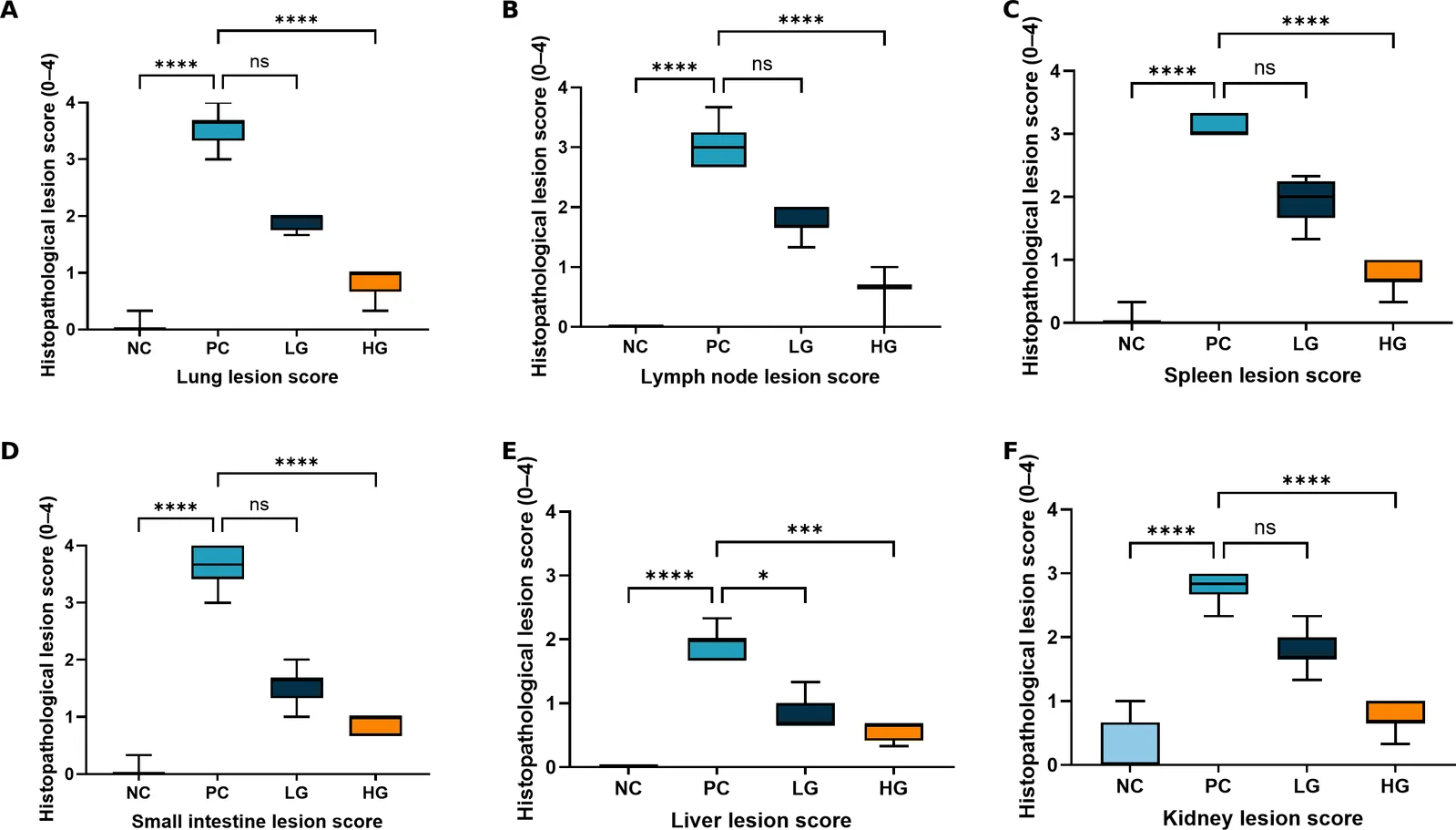
Histopathological lesion scores of major organs at 21 dpi. Lesion scores for the lung (**A**), lymph node (**B**), spleen (**C**), small intestine (**D**), liver (**E**), and kidney (**F**) were evaluated using a semi-quantitative 0–4 ordinal scale, where 0 indicated no obvious lesion and 4 indicated severe lesion. For each piglet, three randomly selected non-overlapping microscopic fields from each organ were scored, and the mean value was used as the final lesion score for that organ. Data are presented as box-and-whisker plots showing the median, interquartile range, and minimum-to-maximum values (*n* = 12 piglets per group). Groups: NC, negative control; PC, PRRSV-infected positive control; LG, low-dose CGA–BAI treatment; HG, high-dose CGA–BAI treatment. Statistical differences were analyzed using the Kruskal–Wallis test, followed by Dunn’s multiple-comparison test. Significance annotations indicate adjusted *P* values: ns, not significant; *P < 0.05; ***P < 0.001; and ****P < 0.0001.

To quantitatively evaluate tissue injury, histopathological lesion scores were assessed using a semi-quantitative 0–4 ordinal scale. For each piglet, three randomly selected non-overlapping microscopic fields from each organ were scored, and the mean value was used as the final lesion score for that organ. Quantitative scoring showed that the PC group had the highest lesion scores in the lung, lymph node, spleen, small intestine, liver, and kidney. The LG group showed an intermediate reduction in lesion burden, whereas the HG group exhibited the lowest lesion scores among PRRSV-challenged groups. Lesion scores were analyzed using the Kruskal–Wallis test followed by Dunn’s multiple-comparison test. These results indicate that high-dose chlorogenic acid-baicalin treatment markedly alleviated PRRSV-induced multi-organ pathological injury (Fig. 5).

### Intestinal microbiota

**Fig 6:**
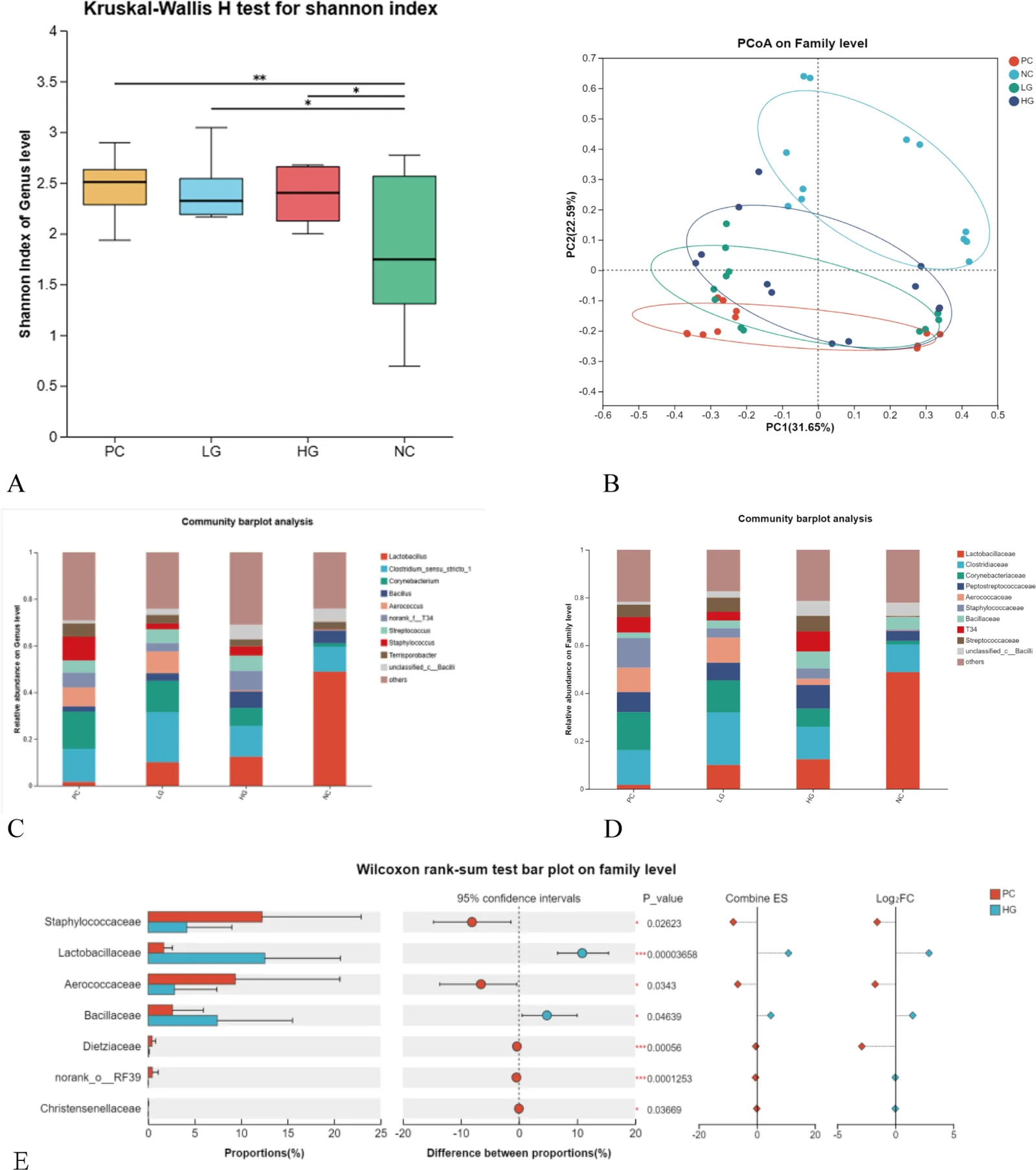
CGA–BAI treatment reshapes the gut microbiota in PRRSV-infected piglets. (**A**) Genus-level Shannon index (box-and-whisker) with the Kruskal-Wallis *H*-test; *P < 0.05, **P < 0.01. (**B**) Principal coordinate analysis (PCoA) based on Bray–Curtis distances at family level; ellipses denote 95% confidence limits. (**C**) Genus-level relative-abundance barplot. (**D**) Family-level relative-abundance barplot. (**E**) Wilcoxon rank-sum test barplot (PC vs HG) at family level: left—proportional difference (%); middle—combined effect size; right—log_2_ fold change. Red = PC, blue = HG. Groups: NC, negative control; PC, positive control; LG, low-dose group; HG, high-dose group.

16S rRNA sequencing revealed that the α-diversity of the gut microbiota in the PC group was significantly reduced (*P* < 0.01), with an increase in opportunistic pathogens and a decrease in Lactobacillus. The microbiota in the LG group partially recovered, while the microbiota profile of the HG group resembled that of the NC group. Compared to the PC group, the HG group showed a significant increase in the relative abundance of Lactobacillus/Lactobacillaceae and a decrease in Clostridiaceae, Peptostreptococcaceae, and Staphylococcaceae (*P* < 0.05), along with enrichment of health-associated taxa, such as the Christensenellaceae family. These results indicate that the CGA–BAI combination was associated with partial restoration of gut microbial composition in PRRSV-infected piglets, with the HG group showing the most pronounced shift toward the NC microbial profile (Fig. 6) .

## DISCUSSION

In this study, through clinical indices, virological assays, immunological evaluation, pathological observation, and gut microbiome analysis, we evaluated the multi-dimensional protective effects of a CGA–BAI compound in weaned piglets infected with PRRSV. Overall, the HG group generally showed greater improvements than the LG and PC groups in clinical, virological, immunological, pathological, and microbiota-related outcomes, suggesting a possible dose-related trend.

The high fever and anorexia caused by PRRSV infection are direct contributors to production losses (1, 3, 4). In this study, the PC group showed sustained fever from 2 to 5 dpi and growth stagnation, whereas the HG group showed only a mild, transient fever, peaking at 3 dpi and returning to the normal range by 6 dpi. At 21 dpi, body weight in the HG group was not significantly different from that in the NC group. Previous studies have suggested that CGA may modulate inflammatory pathways such as COX-2/PGE₂, NF-κB, and MAPK (9, 10), whereas BAI has been reported to interfere with PRRSV replication-related processes *in vitro* (23). These previously reported activities may partly explain the improvements observed here, but these pathways were not directly examined in the present study.

Serum PRRSV RNA levels were significantly lower in the HG group than in the PC group at 7 dpi. Serum PRRSV RNA fell below the qPCR detection limit in the HG group by 21 dpi. Studies have found that CGA can inhibit the release of porcine coronaviruses by targeting viral envelope protein-mediated egress (16), suggesting it may have a similar effect on PRRSV. Previous studies have reported anti-PRRSV activities of both baicalein and baicalin (23, 24). These mechanisms may partly explain the reduced serum PRRSV RNA observed in the HG group, but viral replication proteins and related signaling pathways were not directly examined in the present study.

The course of PRRSV infection is closely tied to an imbalance of “high inflammation and low antiviral response” (25–28). In this study, both LG and HG groups significantly reduced TNF-α, IL-1β, and IL-6 levels while increasing IFN-γ and IL-2, and at the same time maintained IL-10 at a higher level—the latter can buffer immune-mediated damage without suppressing cellular immunity. Previous studies indicate that CGA may activate the Nrf2-HO-1 pathway and inhibit TLR4/NF-κB signaling (29, 30), whereas BAI has been reported to modulate inflammatory responses in macrophages (12). These previously reported mechanisms may be consistent with the cytokine changes observed here.

The PC group exhibited severe lesions such as alveolar destruction, hepatocyte necrosis, and renal tubular injury, whereas these pathological damages were significantly alleviated in the HG group. PRRSV infection has been associated with alterations in the porcine gut microbiota, and these microbial changes may be linked to disease severity and systemic immune responses (8, 31, 32). In this study, the HG group was significantly enriched in Lactobacillaceae, Christensenellaceae, and the order RF39, while it reduced opportunistic taxa such as Staphylococcaceae and Clostridiaceae, and partially restored α-diversity. Previous studies suggest that CGA may support intestinal barrier integrity and modulate the expression of tight junction-related proteins, including ZO-1 and occludin (29, 30); previous piglet studies have shown that BAI supplementation can alleviate intestinal inflammation and microbial disturbances and may promote Lactobacillus colonization (30, 33). Therefore, we speculate that the CGA–BAI combination alleviates multi-organ damage through a cascade mechanism of “antiviral action → barrier stabilization → microbiota modulation → organ protection.”

The HG group generally showed greater improvements than the LG group, suggesting a possible dose-related trend. The oral disposition of both CGA and BAI is influenced by intestinal absorption, metabolism, and gut microbial biotransformation, highlighting the need for dedicated pharmacokinetic studies of their combined administration (34, 35). Both CGA and BAI have been reported to possess anti-inflammatory, antioxidant, antiviral, and immunomodulatory activities. Their combined administration may therefore influence multiple biological responses; however, the present experimental design did not allow the individual contribution of each compound or their potential interaction to be determined. Future studies including CGA-only and BAI-only treatment groups are needed to distinguish their respective effects and evaluate whether the combination produces additive or synergistic activity.

Given the challenges of high PRRSV variability, inconsistent vaccine protection, and interference from maternal antibodies, natural plant bioactive compounds offer a new option for “antibiotic-free, low-residue” disease control. These findings suggest that CGA–BAI supplementation may be a promising adjunctive nutritional strategy. However, its practical application requires validation using contemporary field strains, long-term production trials, and safety and pharmacokinetic evaluations. In combination with biosecurity measures, vaccination, and precision nutrition strategies, this approach could contribute to an integrated PRRSV control system (36).

This study has several limitations. First, only one China-origin PRRSV-2 strain was used, and the findings therefore cannot be directly extrapolated to highly pathogenic, NADC30-like, or currently circulating recombinant PRRSV variants. Second, the follow-up period ended at 21 dpi and was insufficient to assess long-term immune responses and production performance. Third, key molecular pathways, including NF-κB/MAPK, COX-2/PGE₂, tight junction proteins, and PRRSV replication-related proteins, were not directly measured; therefore, the proposed mechanisms remain inferential. In addition, because CGA-only and BAI-only groups were not included, the individual contributions of each compound and any additive or synergistic interactions could not be determined. Future studies should incorporate contemporary field strains, longer follow-up periods, single-compound controls, and targeted molecular or multi-omics analyses to clarify efficacy, mechanism, and optimal dosing. Overall, the high-dose CGA–BAI combination was associated with reduced serum PRRSV RNA load, improved cytokine balance, partial restoration of gut microbial composition, and alleviation of multi-organ lesions in this PRRSV-2 infection model.

### Conclusion

This study evaluated the effects of combined CGA and BAI supplementation in PRRSV-infected weaned piglets. The high-dose combination accelerated fever resolution, improved body weight gain, reduced serum PRRSV RNA to below the qPCR detection limit by 21 dpi, attenuated pro-inflammatory cytokine responses, and increased antiviral cytokine levels. It also alleviated gross and histopathological lesions and partially restored gut microbial composition. These findings suggest that CGA–BAI supplementation may represent a promising adjunct nutritional strategy for mitigating PRRSV-associated disease. Further studies are required to validate its efficacy against contemporary PRRSV variants, clarify molecular mechanisms, optimize dosing, and assess long-term safety and production benefits.

## Data Availability

The raw 16S rRNA gene amplicon sequencing data generated in this study have been deposited in the NCBI Sequence Read Archive under BioProject accession number PRJNA1492574. Other data generated or analyzed during this study are included in this article and its supplementary materials.
